# Emergency Laparoscopic Transabdominal Preperitoneal (TAPP) Repair With Bowel Resection for a Strangulated Femoral Hernia: A Case Report and Review of Strategies

**DOI:** 10.7759/cureus.100063

**Published:** 2025-12-25

**Authors:** Chih-kai Huang, Ching-ming Kwok, Chuan-hsun Chang

**Affiliations:** 1 Division of General Surgery, Department of Surgery, Cheng Hsin General Hospital, Taipei, TWN

**Keywords:** bowel ischemia, decision algorithm, emergency surgery, femoral hernia, laparoscopic tapp, preperitoneal mesh, strangulation

## Abstract

Femoral hernias are uncommon but carry a high risk of incarceration and strangulation. While laparoscopy is increasingly applied in elective groin hernia repair, its role in emergency settings, requiring bowel resection, remains underreported.

We report a 73-year-old woman (BMI 25.8) presenting with acute abdominal pain and vomiting. Examination revealed an irreducible right groin bulge, without overlying skin necrosis. Laboratory tests showed leukocytosis, with a markedly elevated neutrophil-to-lymphocyte ratio (NLR) of 10.7. CT demonstrated a small bowel loop herniating through the right femoral canal, with a mesenteric whirl sign. Diagnostic laparoscopy confirmed incarcerated ileum with a 0.5 cm perforation. Approximately 5 cm of bowel was resected extracorporeally via a mini-laparotomy, followed by transabdominal preperitoneal (TAPP) mesh repair. The patient recovered uneventfully and was discharged on postoperative day 11.

Laparoscopic TAPP allowed safe reduction, assessment of bowel viability, correction of mesenteric torsion, and durable hernia repair within the same setting. Current evidence supports mesh use in selected clean-contaminated fields, and our case demonstrates its feasibility, even when limited bowel resection is required.

Emergency laparoscopic TAPP repair, with concomitant bowel resection, is a safe and effective option for strangulated femoral hernias, offering both diagnostic and therapeutic advantages while minimizing wound morbidity.

## Introduction

Femoral hernias are an uncommon subtype of abdominal wall hernia, accounting for approximately 5% of cases [1]. They occur when abdominal viscera or omentum passes through the femoral ring into the femoral canal, a narrow anatomical compartment bordered by the lacunar ligament medially and by the pectineal (Cooper’s) ligament on the pubis posteriorly. This rigid anatomy makes femoral hernias particularly prone to incarceration and strangulation [2], distinguishing them from inguinal hernias. The condition is more common in women, with a female-to-male ratio of nearly 3:1 - largely due to the wider bony pelvis - and is rarely encountered in children [3]. Reported risk factors include multiparity, advanced age, and chronically elevated intra-abdominal pressure [4].

Clinically, femoral hernias often present as a groin lump, but their deep location can make diagnosis challenging, and misclassification as inguinal hernias is common. Importantly, up to 30% present as emergencies with obstruction or strangulation, and the risk of strangulation rises significantly over time [5]. Once strangulated, femoral hernias may rapidly progress to ischemia, perforation, and sepsis, necessitating urgent surgery.

Traditionally, emergency repair has been performed via open approaches, often avoiding mesh in contaminated fields, though this increases recurrence. Despite the increasing popularity of minimally invasive surgery, laparoscopic management of strangulated femoral hernias remains underreported and lacks consensus guidelines, especially in emergency settings [6]. The role of laparoscopy, particularly transabdominal preperitoneal (TAPP) repair, offers the advantage of intra-abdominal assessment and durable hernia repair [7].

In this report, we describe a case of an acutely incarcerated femoral hernia managed with laparoscopic TAPP repair and bowel resection, accompanied by a literature review.

## Case presentation

**Figure 1:**
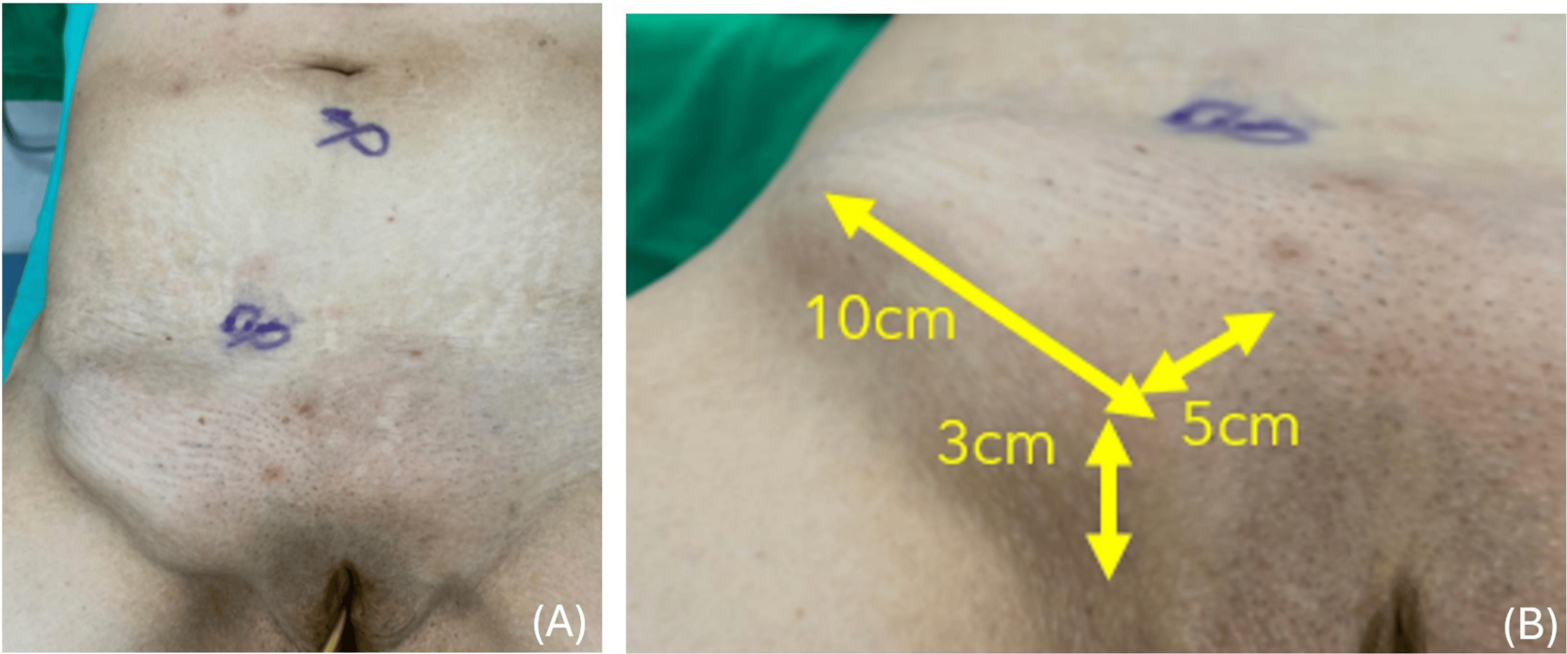
Preoperative clinical findings of the incarcerated femoral hernia (A) Lower abdomen and groin examination shows a bulging mass in the right groin, with preoperative port-site markings for laparoscopic repair. (B) Closer view with measurements demonstrates a bulge measuring approximately 10 × 5 cm, located about 3 cm above the pubic region.

**Figure 2:**
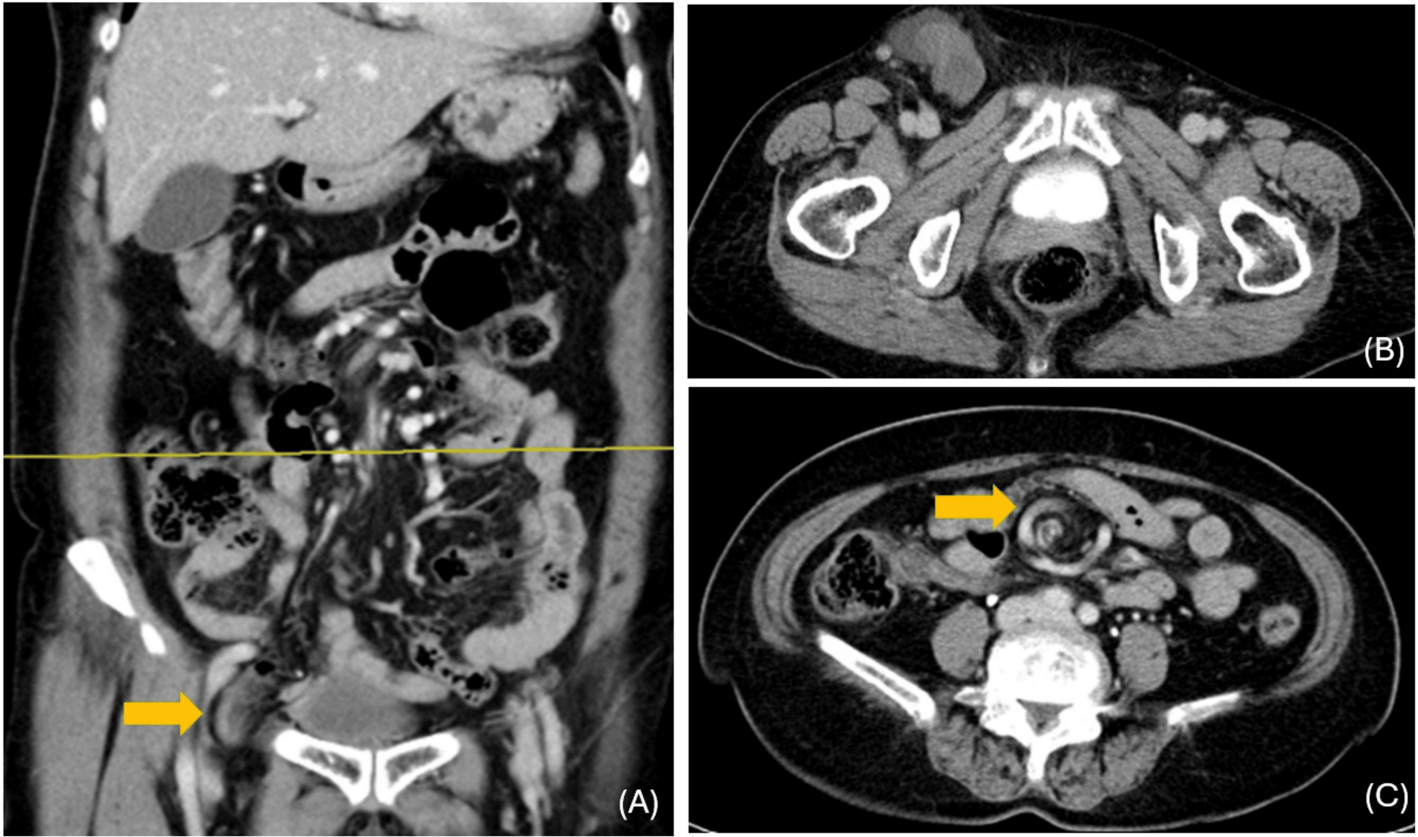
CT findings of an incarcerated femoral hernia (A) Coronal view demonstrates herniation of a small-bowel loop below the inguinal ligament into the right femoral canal (yellow arrow), confirming an incarcerated femoral hernia. (B) Axial pelvic CT at the level of the femoral canal shows a small bowel loop protruding medial to the right femoral vein, consistent with an incarcerated femoral hernia. (C) Axial view shows a mesenteric “whirl sign” (yellow arrow), consistent with volvulus of the small bowel in the setting of groin hernia incarceration.

A 73-year-old previously healthy female (BMI 25.8), with a self-reported lower abdominal hernia for more than three years, presented with a one-day history of lower abdominal pain and vomiting. Physical examination revealed a firm, tender, irreducible bulge measuring approximately 10 × 5 cm in the right groin, located 3 cm above the pubic region and inferior to the inguinal ligament. The overlying skin appeared tense but without necrosis or ulceration (Figures 1A-1B). Laboratory tests showed leukocytosis, with a markedly elevated neutrophil-to-lymphocyte ratio (NLR 10.7). Although C-reactive protein (CRP) was very low (<0.06 mg/dL) at presentation, early normal or low CRP does not exclude strangulation (Table 1). CT demonstrated a small bowel loop herniating through the right femoral canal, with a mesenteric whirl sign, consistent with an incarcerated femoral hernia and raising concern for strangulation (Figures 2A-2C). The interval from emergency department arrival to incision was approximately eight hours.

**Table 1:** Admission laboratory values at ED presentation WBC was elevated at 12.4 × 10³/µL, with marked neutrophil predominance (NEUT% 88.1) and relative lymphopenia (LYMPH% 8.2), yielding an NLR of 10.7 - consistent with a high physiologic stress state in the context of suspected strangulation. WBC, White Blood Cell Count; NEUT%, Neutrophil Percentage; LYMPH%, Lymphocyte Percentage; BUN, Blood Urea Nitrogen; CPK, Creatine Phosphokinase; CRP, C-Reactive Protein; NLR, Neutrophil-to-Lymphocyte Ratio

Test	Result	Units	Reference range	Interpretation
WBC	12.4	10^3/µL	4.0-10.0	Leukocytosis
NEUT%	88.1	%	40.0-75.0	Neutrophilia
LYMPH%	8.2	%	20.0-45.0	Relative lymphopenia
BUN	13.2	mg/dL	6.0-20.0	Within reference
Creatinine	0.51	mg/dL	0.50-0.90	Preserved renal function
CPK	106	U/L	26-192	Within reference
CRP	<0.06	mg/dL	<0.5	Very low
NLR	10.7	Ratio	≈1-3 (laboratory-specific)	Markedly elevated; high physiologic stress

Perioperative care bundle

Pre-operative

On admission, the patient received IV crystalloid resuscitation, multimodal analgesia, and antiemetics, and correction of electrolyte imbalance. Single-agent broad-spectrum prophylaxis was administered, together with mechanical venous thromboembolism prophylaxis. Informed consent included the possibility of conversion to open surgery and temporary stoma formation.

Intra-operative

A laparoscopic TAPP approach was used. Hernia contents were reduced atraumatically, and bowel viability was assessed by direct inspection ± indocyanine green (ICG) fluorescence, when available. Non-viable bowel was managed by extracorporeal segmental resection via a 3- to 5-cm peri-umbilical mini-laparotomy with stapled side-to-side anastomosis. The fascial defect was then closed to a trocar-sized opening. Contamination control included copious intraperitoneal irrigation, glove change, and a clean instrument set for the preperitoneal phase. The peritoneal flap was elevated in a quadrant remote from the mini-laparotomy. The macroporous polypropylene mesh was placed in the preperitoneal space with suture fixation, and the peritoneal flap was securely closed.

Post-operative

An ERAS (Enhanced Recovery After Surgery) pathway was followed: early ambulation and advancement of oral intake as tolerated, with multimodal opioid-sparing analgesia. The wound was reviewed at 24-48 hours, and outpatient follow-up was arranged at 30-90 days; targeted ultrasound or CT was planned if clinical concern for recurrence arose.

Operative findings

**Figure 3:**
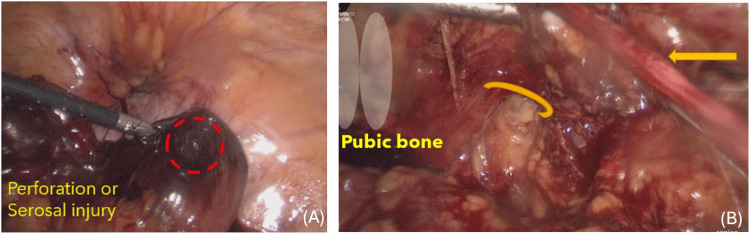
Intraoperative laparoscopic findings and laparoscopic view in the preperitoneal space (A) A dusky, congested loop of small bowel was identified, consistent with compromised viability due to femoral hernia incarceration. A focal serosal defect with possible impending perforation is seen at the antimesenteric border (dashed red circle). (B) The femoral canal (curved arrow) is exposed adjacent to the pubic bone (labeled). The round ligament (straight arrow) is identified superiorly.

Diagnostic laparoscopy revealed a segment of ileum incarcerated at the femoral canal. The loop was reduced and appeared dusky, with a 0.5 cm perforation, necessitating resection of approximately 5 cm of bowel located 50 cm proximal to the ileocecal valve. Mesenteric torsion was identified and corrected. Bowel continuity was restored extracorporeally through a 3-cm periumbilical mini-laparotomy, after which definitive hernia repair was performed via a TAPP approach using a macroporous polypropylene mesh (Optilene) (Figures 3A-3B). The patient’s postoperative recovery was uneventful, with oral intake resumed by day 4 and discharge on day 11.

## Discussion

In the presented case, a TAPP laparoscopic approach was intentionally selected over the conventional anterior transinguinal route to allow for comprehensive intra-abdominal inspection and simultaneous management of the incarcerated bowel. This minimally invasive technique permitted safe reduction of the herniated loop, thorough assessment of bowel viability, and definitive hernia repair within the same setting. A limited midline mini-laparotomy was added solely to facilitate extracorporeal bowel resection and anastomosis. By avoiding a groin incision, the patient benefited from improved postoperative wound monitoring and a potentially reduced risk of wound-related complications. This strategy underscores the diagnostic and therapeutic versatility of TAPP in emergency hernia management and its role in reducing groin morbidity.

Despite the presence of a small perforation, intraoperative contamination was well controlled via an intraoperative care bundle, and the use of synthetic mesh in the preperitoneal space was deemed appropriate. Mounting evidence supports this approach. A 2023 meta-analysis by Marcolin et al. demonstrated that mesh repair significantly reduces hernia recurrence (OR 0.36, 95% CI: 0.19-0.67; p = 0.001), with only a modest increase in surgical site infections in patients undergoing bowel resection (OR 1.74; p = 0.04) [8]. Similarly, the 2017 World Society of Emergency Surgery (WSES) guidelines recommend mesh usage even in selected clean-contaminated settings, citing no increase in 30-day wound-related morbidity [9]. Retrospective data by Emile et al. (2017) show comparable infection rates between mesh and non-mesh repairs, with lower recurrence in the mesh group [10]. Smith et al. (2021) further corroborated the safety of laparoscopic TAPP repair in strangulated hernias requiring bowel resection, reporting no significant increase in mesh infection or early recurrence [11]. Overall, these findings support the feasibility of mesh implantation in carefully selected clean-contaminated fields, particularly when placement is deferred until after resection has been completed via a separate incision.

From a technical standpoint, closure of the peritoneal flap during TAPP repair is a critical step to prevent direct mesh contact with intra-abdominal viscera [12]. Failure to achieve secure closure may predispose to complications such as adhesion formation, mesh erosion, or enterocutaneous fistula [13]. In emergency settings, this step can be particularly challenging due to tissue edema or friability. Proficiency in laparoscopic suturing is essential to minimize these risks and ensure durable mesh fixation. The necessity of advanced intracorporeal suturing skills further highlights the need for surgical expertise when performing TAPP in complex, emergency scenarios [14].

While laparoscopy was successfully employed in this case, the modified McEvedy high approach remains a valuable alternative in selected patients. It provides direct preperitoneal access, facilitates safe reduction of the hernia sac, and allows bowel resection when necessary. Additionally, it avoids disruption of the inguinal canal, preserving structural integrity and reducing the risk of long-term morbidity associated with open transinguinal approaches [15]. This approach offers a sound, open surgical alternative when laparoscopy is contraindicated or unavailable.

The patient’s markedly elevated NLR of 10.7 exceeded the threshold range of ~1-3 in healthy adults. This biomarker has been proposed as a useful adjunct in identifying strangulated hernias requiring urgent intervention. Despite normal CRP and creatine phosphokinase (CPK) levels, the elevated NLR in conjunction with clinical features of obstruction reinforced the need for prompt surgical exploration rather than attempted manual reduction. These findings align with emerging evidence supporting NLR as an early predictor of ischemic insult in incarcerated hernias [16].

To provide further perspective, a contemporaneous case involving an incarcerated inguinoscrotal hernia was managed by conventional open exploration. The hernia sac contained a segment of ischemic transverse colon, and, due to the concern for contamination and uncertain bowel viability, a non-mesh Bassini repair with diverting loop colostomy was performed. This case illustrates the rationale for avoiding prosthetic material in clearly contaminated fields and highlights the traditional open approach as a still-relevant strategy. It also provides a useful contrast to the laparoscopic management strategy employed in our patient.

**Figure 4:**
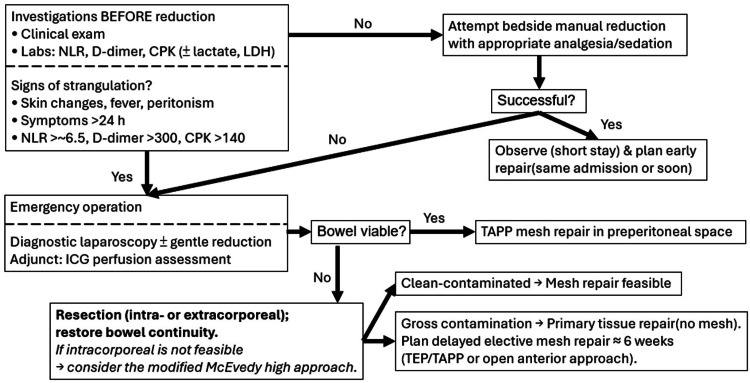
Pragmatic option-based pathway for irreducible groin hernia in selected patients NLR, Neutrophil-to-Lymphocyte Ratio; CPK, Creatine Phosphokinase; LDH, Lactate Dehydrogenase; ICG, Indocyanine Green; TEP/TAPP, Totally Extraperitoneal repair/Transabdominal Preperitoneal repair

Based on current evidence and our experience, we propose an option-based pathway for irreducible groin hernia in selected, hemodynamically stable patients in centers with laparo-endoscopic expertise and ICG capability (Figure 4). Before any bedside reduction, perform a clinical assessment and laboratory tests to screen for strangulation [17]. If strangulation is suspected, proceed directly to emergency operation. If not suspected, attempt gentle taxis [18]; when successful, observe briefly and schedule early repair. Intraoperatively, diagnostic laparoscopy, with optional indocyanine-green perfusion assessment, guides management [19]: if the bowel is viable, perform TAPP mesh repair; if non-viable, resect and restore continuity. In hospitals where ICG is unavailable, management should rely on the surgeon's expertise, with the NLR as an adjunct; when bowel strangulation is suspected, resection and anastomosis are prioritized. Mesh repair can be safe in selected patients, whereas gross contamination warrants primary tissue repair without mesh, and delayed elective totally extraperitoneal repair (TEP)/TAPP at about six weeks [20].

## Conclusions

Emergency repair of incarcerated or strangulated groin hernias requires rapid intervention, timely assessment of bowel viability, and strategies intended to reduce recurrence risk. In this case, laparoscopic TAPP with extracorporeal limited resection was feasible and uneventful in a hemodynamically stable patient within a clean-contaminated (no gross spillage) field, after contamination control and secure peritoneal closure; this approach can be performed safely in carefully selected patients by experienced teams, and where resources (±ICG) are available, offering diagnostic and therapeutic advantages with potentially lower wound morbidity.

Conversely, when gross contamination or physiologic instability is present, open non-mesh repair, with or without diversion, remains appropriate, underscoring individualized decision-making. Current guidelines and series support mesh use in clean and selected clean-contaminated settings, but the evidence is heterogeneous and often short-term. Our follow-up (30-90 days) is limited; thus, these observations are preliminary and require confirmation. Prospective, adequately powered studies are needed to refine patient selection, contamination thresholds, and the role of laparoscopy - particularly when concomitant bowel resection is required - in emergency groin hernia repair.
